# The Remineralizing and Desensitizing Potential of Hydroxyapatite in Dentistry: A Narrative Review of Recent Clinical Evidence

**DOI:** 10.3390/jfb16090325

**Published:** 2025-09-04

**Authors:** Jusef Naim, Sinan Sen

**Affiliations:** Department of Orthodontics, University Hospital of Schleswig-Holstein, 24105 Kiel, Germany; sinan.sen@uksh.de

**Keywords:** hydroxyapatite, fluorides, caries prevention, remineralization, dentin sensitivity, biocompatible materials, molar–incisor hypomineralization, molar hypomineralization

## Abstract

Although caries is declining in industrialized countries, early childhood caries and molar–incisor hypomineralization (MIH) remain clinically relevant. To meet the demand for effective and well-tolerated preventive strategies, hydroxyapatite (HAp) has gained attention as a biocompatible, fluoride-free agent. A structured narrative review was conducted to evaluate recent clinical evidence on the use of HAp. A PubMed search identified clinical trials from the past five years that investigated HAp-based products. Studies were included if they reported clinical outcomes related to remineralization, caries prevention, or desensitization. Fifteen clinical studies met the inclusion criteria. HAp seems to be a safe and effective alternative to flouride, especially for children or individuals at risk of overexposure. While both agents show similar efficacy in caries prevention, HAp may offer additional advantages in managing hypersensitivity and MIH. Compared to other remineralizing agents, such as calcium sodium phosphosilicate, HAp demonstrated comparable efficacy. Combination therapies show the most promising results. Future research should explore synergies of active ingredients and include standardized long-term studies to substantiate the clinical relevance of HAp.

## 1. Introduction

Although caries prevalence has been declining in highly developed countries such as Germany [1], early childhood caries remains a persistent global health issue [2]. In addition to caries, molar–incisor hypomineralization (MIH) has increasingly become a focus of research. In 2003, the European Academy of Paediatric Dentistry first defined this condition as an etiologically unclear enamel mineralization disorder that can affect one or more first permanent molars as well as incisors [3]. The severity of defects ranges from mild opacities to pronounced structural loss, often accompanied by hypersensitivity, pain, and functional problems, which increase the susceptibility to caries [4].

The treatment of carious lesions or lesions of other origins has fundamentally changed over the past decades. While extensive excavation of carious areas was once common practice, modern dentistry is increasingly guided by minimally invasive treatment concepts, which in some cases extend to infiltration techniques for hypomineralized areas [5].

Considering this background, prevention plays a central role. Thanks to extensive educational campaigns [6], ongoing research, and the development of innovative products, significant progress has been made in caries prevention.

The use of fluoride (Fl) is still considered the gold standard in this context [7]. Histologically, dental enamel consists of prisms made up of clusters of tiny crystallites. The spaces between them contain water and organic material, forming diffusion pathways for acids, minerals, and fluoride ions [8]. Fl is incorporated into the enamel’s apatite structure via ionic exchange, where fluoride ions (F^−^) replace hydroxide ions (OH^−^), resulting in a more stable and acid-resistant crystal lattice. Another key fluoride mechanism is the formation of a calcium fluoride (CaF_2_)-like layer on the enamel surface, which serves as a reservoir. Under acidic conditions, it dissolves and releases fluoride, promoting remineralization [9,10]. However, Fl does not enjoy universal acceptance, especially due to concerns about potential side effects from improper use and the risk of dental fluorosis [11].

In recent years, Fl-free or Fl-reduced alternatives have gained increasing importance. These include, in particular, casein phosphopeptide–amorphous calcium phosphate (CPP-ACP) [12] and hydroxyapatite (HAp) [13], both of which exhibit high biocompatibility and have the potential to actively promote remineralization. For teeth affected by MIH, these agents have also shown promising results [14,15].

The first scientific studies on HAp in dentistry date back to the 1980s and were initially conducted in Japan [16]. In 2006, the first biomimetic, synthetic toothpaste containing HAp as a remineralizing agent was introduced to the European market [17].

**Figure 1:**
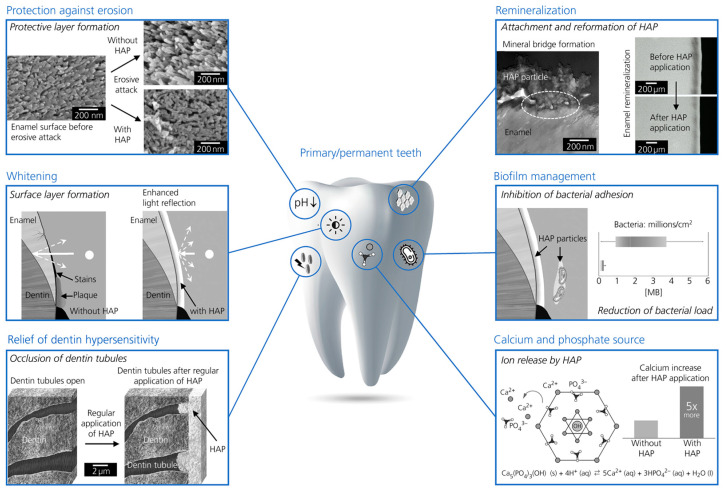
Key modes of action of hydroxyapatite in the prevention and treatment of oral diseases. Reprinted from Ref. [33].

Hydroxyapatite (Ca_5_(PO_4_)_3_(OH)) is a crystalline calcium phosphate that naturally occurs in teeth and bones [18]. This structural and chemical similarity to human enamel gives HAp biomimetic properties, making it particularly attractive as an active ingredient in preventive dentistry. It has been demonstrated that HAp particles can adhere to damaged enamel and fill microscopic defects, thereby restoring surface integrity [19,20]. In particular, 20 nm-sized HAp particles show high affinity to the natural enamel structure in vitro, whereas larger particles appear to be less effective [21]. The nanoscale size of HAp increases its surface area, enhancing adhesion and enabling the repair of microdefects on the enamel surface [17]. HAp also exhibits clinically relevant effects in cases of dentin hypersensitivity by penetrating the dentinal tubules, occluding them, and thus reducing fluid movement, a mechanism considered a major cause of hypersensitivity [22]. Since enamel is unable to regenerate itself, the exogenous application of HAp represents a promising approach to support remineralization. The different mechanisms of action of HAp in the prevention and treatment of oral disease are illustrated in Figure 1.

Beyond conservative dentistry, HAp is also used in other fields, such as orthopedics [23], oral surgery, e.g., for implant coatings [24,25] or as a bone substitute material [26]. In endodontics, HAp is being experimentally investigated for the regeneration of pulp-sensitive structures [27].

In daily practice, HAp is currently available in various formulations, including toothpastes [28], gels [29], mouthwashes [30] and chewing gums [31], each in varying concentrations [32].

Although Fl remains the gold standard, its biocompatibility, particularly at higher concentrations, has been subject to increasing criticism. As a result, biomimetic alternatives such as HAp are gaining growing interest in current research. This narrative review aims to provide a comprehensive overview of recent clinical studies investigating the use of HAp in the context of remineralization, caries prevention, and the reduction in dentin hypersensitivity. The review seeks to evaluate the clinical efficacy of HAp based on the available evidence and to position it in the context of other active agents. Given that HAp has not yet been widely adopted in clinical practice and the current evidence base remains partly heterogeneous in in vitro studies, a relevant research gap becomes apparent. Therefore, this review aims to summarize the current state of research and to outline potential directions for the future development and clinical application of HAp.

**Research Question**

This narrative review aims to provide an up-to-date overview of recent clinical studies investigating the use of HAp. The focus lies on its potential to promote hard tissue remineralization, prevent caries and reduce dentin hypersensitivity. The central research question is: What is the current clinical evidence regarding the remineralizing, caries-preventive and desensitizing effects of HAp in dental applications?

**Search strategy**

A structured literature search was conducted using the PubMed database to identify relevant clinical studies on the use of HAp. The search term “hydroxyapatite AND dentistry” was used, with filters applied to include only clinical trials published within the past five years.

The PICO framework was defined as follows:

- P (Population): Children and adults receiving non-invasive dental care for the purposes of remineralization, caries prevention, or management of dentin hypersensitivity.
- I (Intervention): Hydroxyapatite-containing products (e.g., toothpaste, gel, lotion, chewing gum).
- C (Comparison): Alternative agents, placebo, or no intervention.
- O (Outcome): Improvement in enamel or dentin remineralization, reduction in caries incidence, or decrease in dentin hypersensitivity.

Eligible studies investigated HAp-containing products (e.g., toothpaste, gel, lotion, or chewing gum) applied in non-surgical dental settings. Clinical endpoints had to relate to enamel or dentin remineralization, caries prevention, or a reduction in hypersensitivity. An initial title and abstract screening was conducted to identify relevant articles according to predefined inclusion criteria. Studies outside the dental field, as well as those focusing on oral surgical applications, were excluded at this stage.

In the second phase, a full-text analysis was performed to assess methodological and content-related eligibility. Studies were included if they reported clinical outcomes quantified either through standardized indices (e.g., ICDAS, SAI, PI, BEWE) or via instrumental methods such as radiographic imaging, microhardness testing, or elemental analysis (e.g., Ca/P ratio). Following content evaluation, all included studies were systematically summarized in tabular form.

The final search was completed on 28 February 2025.

## 2. Literature Search and Study Selection

**Figure 2:**
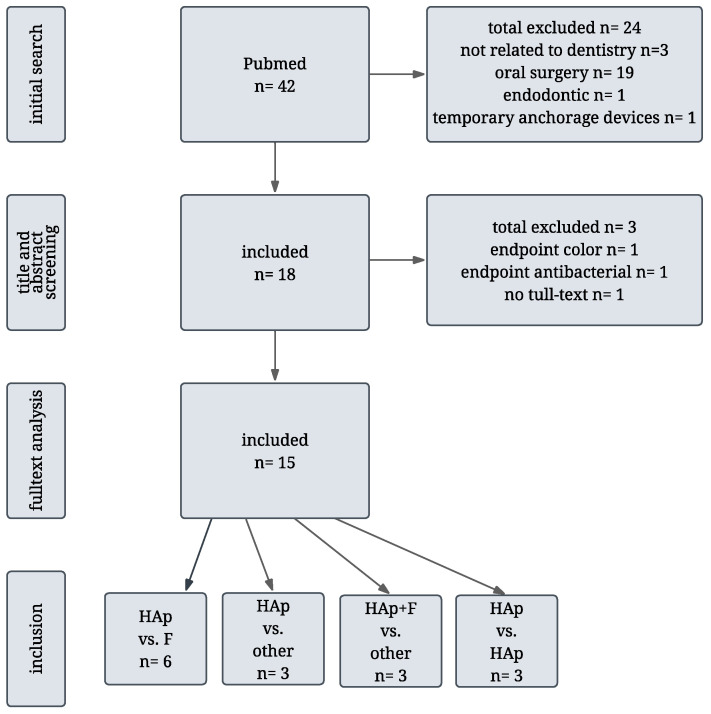
Modified PRISMA flow diagram illustrating the literature selection process in this narrative review.

The initial literature search yielded a total of 42 results. Three studies were excluded due to a lack of relevance to dentistry (two related to maxillofacial surgery and one to orthopedics). An additional 19 studies focused on HAp in the context of oral surgery and were excluded for not meeting the inclusion criteria. One study addressing endodontic applications was also excluded. Furthermore, four studies were related to orthodontics, one of these was excluded during the title and abstract screening due to its focus on temporary anchorage devices. Ultimately, 18 studies were included in the full-text analysis. During this phase, three studies were excluded: one due to the lack of full-text availability, one because it assessed only color change as the endpoint without evaluating remineralization, and one that primarily addressed the antibacterial effect of HAp. A total of 15 studies were included in this narrative review. The modified PRISMA flow diagram is shown in Figure 2. All selected studies investigated the remineralization potential, caries-preventive effects, or desensitizing properties of HAp in non-surgical dental applications.

### Overview and Categorization of Included Studies

To enhance comparability, the included studies were categorized into four groups based on the intervention types: Table 1 presents studies comparing HAp with Fl. Table 2 compares HAp with other active agents. Table 3 summarizes studies evaluating HAp in combination with Fl versus Fl alone. Table 4 includes intra-group comparisons of different HAp formulations or application protocols.

**Hydroxyapatite vs. Fluoride**

The comparison between HAp and Fl revealed that both agents demonstrate comparable effectiveness overall. In terms of caries prevention, none of the included studies showed HAp to be inferior to Fl. Findings on the remineralization potential were heterogeneous: while one study reported no measurable remineralizing effect for HAp (but did detect such effects for Fl), another observed slightly superior outcomes with weekly HAp application compared to monthly NaF application in the context of interproximal reduction during orthodontic treatment.

**Hydroxyapatite vs. other agents**

When compared to alternative remineralizing agents, HAp showed similar efficacy to calcium sodium phosphosilicate in both caries prevention and hypersensitivity reduction. Additionally, one study reported that HAp reduced hypersensitivity in MIH cases and improved MIH-Treatment Need Index. In contrast, when compared to ozone therapy, HAp was less effective, but still resulted in a measurable remineralizing effect compared to baseline.

**Hydroxyapatite + Fluoride vs. other**

The combination of HAp and Fl demonstrated clear superiority over Fl alone in terms of caries prevention and remineralization efficacy.

**Hydroxyapatite vs. Hydroxyapatite**

Studies comparing different HAp formulations indicated that higher concentrations of HAp were associated with enhanced remineralization outcomes. Furthermore, synthetic HAp was found to be more effective than plant-based HAp in delivering calcium ions.

## 3. Interpretation and Implications

This narrative review provides an up-to-date overview of the latest clinical evidence regarding the potential of HAp for remineralization, caries prevention, and desensitization and positions it in the context of other active agents. While numerous in vitro studies form the basis of HAp research, their results vary considerably. While Guntermann et al., in their in vitro study, concluded that HAp is inferior to Fl in terms of remineralization [45], Grewal et al. reported the exact opposite—demonstrating a superiority of HAp over Fl [46]. These conflicting findings raise the question of how reliably laboratory results can be translated into clinical practice. They underscore the urgent need for high-quality clinical studies to further investigate the true efficacy of HAp, especially in light of the growing interest in Fl-free alternatives. To ensure reliable conclusions, this review specifically focuses on in situ studies assessing the efficacy of HAp within the natural oral environment. It aims to systematically synthesize and critically evaluate the current clinical evidence on HAp efficacy under clinically relevant conditions.

### 3.1. Comparative Efficacy of Hydroxyapatite and Fluoride

Dental hard tissues are exposed daily to various chemical challenges, particularly from acidic foods and bacterial metabolic products. When the pH level drops below a critical threshold, demineralization of the enamel occurs, resulting in a net loss of calcium and phosphate ions. Remineralization can occur if the oral environment becomes enriched with suitable mineralizing agents, whether through saliva or external sources, allowing them to reintegrate into the enamel structure [13]. Remineralizing agents with caries-preventive potential are becoming increasingly important for patients, further emphasizing the significance of the preventive approach in contemporary dentistry.

Since Fl still represents the gold standard and remains the main active ingredient in most toothpastes, it is of clinical interest to compare its caries-preventive efficacy with that of HAp. With regard to caries prevention, no inferiority of HAp compared to Fl could be demonstrated in the included studies [34,35], which strongly supports the comparable efficacy of both agents for this particular indication. Both studies published by Paszynska et al. are methodologically robust and feature extended observation periods. While the 2023 publication examined adults [34], a highly similar study design was applied in the 2021 trial involving children [35].

These parallels gain further significance when considering specific high-risk groups such as young children. In this age group, accidental ingestion of toothpaste is common, and the recommended dosage of Fl-containing toothpaste is often exceeded, a factor that significantly increases the risk of developing dental fluorosis [47,48,49]. Moreover, the use of Fl is recurrently debated in the scientific literature in the context of potential systemic health effects, including neurological disorders, although there is currently no conclusive scientific evidence to support these concerns [50]. Against this background, it becomes increasingly important to mitigate the potential risks of excessive Fl exposure in early childhood. Based on the results of the aforementioned studies, it may therefore be concluded that in cases where swallowing of toothpaste is likely, the use of HAp instead of Fl should be preferred, particularly in young children. Due to its chemical similarity to natural bone and tooth structures, HAp demonstrates excellent biocompatibility and clinically relevant toxicity can be ruled out [51,52].

In terms of hypersensitivity, HAp outperforms Fl in one study in reducing hypersensitivity associated with white spot lesions [36], another study found a comparable reduction in sensitivity following bleaching with Fl treatment [37]. Despite its clinical design, the study by Gümüştaş et al. [29] presents a key limitation: the remineralizing agents were applied only once prior to the bleaching procedure, and no information was provided about the oral care products used by participants during the seven-day observation period. This methodological gap significantly limits the comparability of the groups. Nevertheless, both Fl and HAp proved to be more effective than the placebo and CPP-ACP groups.

A direct overall comparison between HAp and Fl reveals that, based on the current clinical evidence, no clear superiority of either agent can be established. Several other reviews have reached the same conclusion [32,53].

### 3.2. Hydroxyapatite in the Management of Molar–Incisor Hypomineralization

Given the global prevalence of approximately 14% for MIH, appropriate home care strategies play a pivotal role. Affected patients often suffer from functional impairments [54], pain [55], and/or increased sensitivity [56]. The impact of applying toothpaste two to three times daily to hypomineralized teeth should not be underestimated, as this practice can alleviate symptoms and improve home care of MIH. In this context, HAp shows promising potential. One of the studies included in this review, using a split-mouth design, demonstrated that a nine-month application of zinc-HAp significantly reduced both the MIH Treatment-Need-Index and the hypersensitivity of affected teeth [40]. The remineralizing effect of HAp is further supported by additional in situ studies [11], and other investigations confirm its efficacy in reducing hypersensitivity [57]. This effect is likely due to mineral deposition, which contributes to the formation of a protective layer on the enamel and tooth surface [58]. Pepla et al. and Enax et al. support these findings in their review [17,59].

In addition to HAp, CPP-ACP has also shown promising results [17,60,61]. Future research should aim to systematically investigate the differences between available active agents and assess the conditions under which specific substances are most suitable, or whether combined application protocols may further enhance patient outcomes.

### 3.3. Advantages of Combination Therapies

The combination of HAp and Fl yielded the most promising results regarding remineralization in several studies. In all investigations, combination formulations proved to be more effective than Fl alone [29,42,43]. The observed differences can be attributed to the divergent mechanisms of action of each compound, particularly with regard to their enamel affinity and depth of mineral penetration. Mielczarek et al. expanded conventional research approaches by analyzing the remineralizing effects both in superficial and deeper enamel layers. Their results show that different agent combinations produce different remineralization profiles: while CPP-ACP combined with Fl enhanced mineralization in superficial enamel regions, the combination of HAp and Fl was more effective in deeper layers. This differentiated approach provides a promising foundation for future research [62].

### 3.4. Limitations of the Current Evidence

Despite the promising findings of the included studies, the limitations of this review must also be critically acknowledged. Firstly, the literature search was limited to a single database, which may have resulted in an incomplete selection of relevant studies. Secondly, many of the included studies had relatively short observation periods, restricting the ability to draw conclusions about the long-term efficacy of HAp. Furthermore, the methodological heterogeneity among studies—regarding both the study populations and the formulation and concentration of the HAp used—limits the strength of evidence and currently prevents the derivation of practical clinical recommendations with sufficient certainty. Overall, while the potential of biomimetic HAp appears promising, further standardized and long-term investigations are needed to reliably substantiate its clinical relevance.

## 4. Conclusions

The available evidence remains inconclusive regarding the superiority of HAp over Fl in terms of remineralization efficacy. However, no clear inferiority of HAp has been shown either, particularly in the context of caries prevention. For specific indications such as dentin hypersensitivity and MIH, HAp may even confer distinct clinical advantages.

Given the risk of toothpaste ingestion in young children, HAp represents a safe and promising alternative to Fl. Once ingestion is no longer a concern, combined application of HAp and Fl appears to be a rational strategy with potential synergistic effects. Several studies have demonstrated that combination therapies may outperform monotherapies in achieving superior clinical outcomes.

Despite these encouraging findings, the current evidence base remains constrained by heterogeneity in study design, short observation periods, and the absence of standardized outcome measures. Future research should therefore prioritize long-term randomized controlled trials with standardized protocols. In addition, direct comparative studies of HAp, Fl, and their combinations across different age groups and clinical contexts are needed, as well as mechanistic studies aimed at elucidating the synergistic effects of combined therapies.

In summary, HAp has demonstrated non-inferiority to Fl in caries prevention, shows favorable outcomes in dentin hypersensitivity, and provides clinical benefits in the management of MIH. Combination therapies indicate the potential to be more effective than monotherapies, but the overall clinical relevance of HAp still requires confirmation through rigorous, long-term studies with standardized outcome measures.

## Figures and Tables

**Table 1:** Summary of included studies comparing hydroxyapatite vs. fluoride.

Author and Year	Objective	Methods	Result	Conclusion	Review Comment
Paszynska et al. [34] 2023	Toothpaste comparison: HAp vs. NaF	189 adults; non-inferiority trial in terms of no increase in DMFS over 18 months	89.3% (HAp) vs. 87.4% (NaF) showed no increase in DMFS	HAp is a safe, effective, and non-inferior alternative to NaF	HAp is as effective as NaF in preventing caries
Paszynska et al. [35] 2021	Toothpaste comparison: HAp vs. Fl	207 children; non-inferiority trial in terms of caries progression over 12 months	72.7% (HAp) vs. 74.2% (Fl) showed caries progression; HAp non-inferior to fluoride	HAp is not inferior to Fl in preventing caries in primary dentition	HAp is as effective as Fl in caries prevention
Butera et al. [36] 2022	Toothpaste comparison in white spot lesions: HAp vs. NaF	40 adults; SAI, VAS and BEWE examination over 90 days	SAI and VAS significantly decreased in HAp group; no significant change in BEWE in either group	HAp more effective than NaF in reducing dental hypersensitivity	HAp outperforms NaF in reducing dental hypersensitivity
Gümüştaş et Dikmen [37] 2022	Comparison of HAp (solution) vs. NaF (gel) vs. other before in-office bleaching to reduce sensitivity	64 adults; agent applied before bleaching with 38% H_2_O_2_; sensitivity (VAS) and color change measured	No difference in bleaching efficacy; HAp and NaF significantly reduced sensitivity vs. other	HAp and NaF reduce bleaching-induced sensitivity without affecting whitening effect	HAp as effective as NaF in reducing sensitivity
Wierichs et al. [38] 2020	Toothpaste comparison: HAp vs. F	20 adults; 4 toothpastes (HAp, NaF 0, 1100, 5000 ppm) each tested over 4 weeks using mandibular appliances; mineral loss and lesion depth measured on bovine enamel and dentin	HAp and NaF0 showed demineralization; NaF1100 and NaF5000 showed significant remineralization, especially in highly demineralized dentin	Only NaF toothpastes showed dose-dependent remineralizing effects; HAp was not effective	HAp does not prevent demineralization; only NaF effective in remineralization
Dussa et al. [19] 2024	Remineralization effects after IPR with HAp vs. NaF	25 patients; 5 groups (HAp weekly, NaF monthly, 3 controls); enamel assessed via Ca/P ratio, Vickers microhardness, and surface roughness	HAp resulted in highest remineralization (close to untreated enamel). NaF showed a slightly weaker trend but also promoted enamel remineralization	HAp was slightly more effective than NaF; however, application frequency differed (HAp weekly, NaF monthly)	HAp may be more suitable post-IPR due to frequent application without risk of fluoride overexposure

(HAp: hydroxyapatite, NaF: sodium fluoride, DMFS: decayed, missing, and filled surfaces, Fl: fluoride, SAI: Schiff Air Index, VAS: Visual Analogue Scale, BEWE: Basic Erosive Wear Examination, H_2_O_2_: hydrogen peroxide, IPR: interproximal reduction, Ca/P ratio: calcium-to-phosphorus ratio).

**Table 2:** Summary of included studies comparing hydroxyapatite vs. other agents.

Author and Year	Objective	Methods	Result	Conclusion	Review Comment
Scribante et al. [39] 2024	Toothpaste comparison: zinc-HAp (with monthly HAp gel) vs. calcium sodium phosphosilicate	40 children with asthma/allergic rhinitis; Indices assessed over 6 months: SAI, PI, pH, DMFT, BEWE, SI	SAI and PI significantly reduced in both groups; no significant intergroup differences overall	Both groups showed similar clinical improvement	since calcium sodium phosphosilicate forms HAp, comparable effects are expected
Butera et al. [40] 2022	Evaluate zinc-HAp paste for desensitizing and remineralizing MIH-affected teeth	25 children with MIH lesions; zinc-HAp paste applied to 2 teeth, 2 untreated control teeth (split-mouth); HAp-free toothpaste used in both groups; PCR, MIH-TNI, SAI assessed over 9 months	PCR improved in both groups; SAI and MIH-TNI improved significantly in zinc-HAp group, but showed only minor changes in control group	Zinc-HAp reduced sensitivity and improved MIH-TNI	Zinc-HAp paste shows desensitizing effect and supports remineralization in MIH treatment
Grocholewicz et al. [41] 2020	Compare remineralization of initial approximal caries using HAp gel, ozone therapy, or both	92 adults; all used fluoride toothpaste; 6-month treatment with HAp gel, ozone, or both; follow-up over 2 years	After 1 year: remineralization in 36.5% (HAp), 52.8% (ozone), 69.3% (combined); caries reversal in 18.0%, 38.0%, and 45.4%, respectively, over 2 years	All treatments showed remineralizing effects; combination was most effective but required continued application	HAp + ozone showed best short-term effect; ozone outperforms HAp; long-term benefit depends on consistent use

(HAp: hydroxyapatite; SAI: Schiff Air Index; PI: Plaque Index; DMFT: Decayed, Missing, Filled Teeth; BEWE: Basic Erosive Wear Examination; SI: Susceptibility Index; MIH: molar–incisor hypomineralization; PCR: Plaque Control Record; MIH-TNI: molar–incisor hypomineralization Treatment Need Index).

**Table 3:** Summary of included studies comparing hydroxyapatite + fluoride vs. other agents.

Author and Year	Objective	Methods	Result	Conclusion	Review Comment
Campus et al. [42] 2024	Toothpaste comparison: HAF vs. F	610 children; 4 toothpastes (2 HAF, 2 fluoride); plaque pH and microbiological analyses over 24 months	All groups showed increased minimum pH; HAF had the greatest increase and significantly fewer cariogenic bacteria	HAF toothpaste shows promising effects, but evidence remains inconclusive	HAF may outperform F alone
Cagetti et al. [43] 2022	Toothpaste comparison: HAF vs. Fl in caries prevention	610 children; 4 toothpastes (2 HAF, 2 Fl); ICDAS scoring over 24 months	HAF significantly reduced caries progression in both primary and permanent dentition; risk reduction 38–39%	HAF shows greater risk reduction for caries compared to Fl over 2 years	HAF may outperform Fl alone
Esparza- Villalpando et al. [29] 2021	Topical agent comparison for white spot lesions in primary enamel: Fl + HAp vs. Fl + CPP-ACP vs. NaF	130 children; topical application of Fl + HAp, Fl + CPP-ACP, NaF; UF measured over 3 weeks; regular toothpaste use unknown	UF decreased in all groups; Fl + HAp and Fl + CPP-ACP more effective than NaF; no significant difference between Fl + HAp and Fl + CPP-ACP	Fl + HAp and Fl + CPP-ACP are more effective than NaF in remineralizing	Additives like HAp or CPP-ACP outperform NaF alone

(HAF: hydroxyapatite–fluoride; Fl: fluoride; ICDAS: International Caries Detection and Assessment System; CPP-ACP: casein phosphopeptide–amorphous calcium phosphate; NaF: sodium fluoride; UF: units of fluorescence).

**Table 4:** Summary of included studies comparing hydroxyapatite vs. hydroxyapatite.

Author and Year	Objective	Methods	Result	Conclusion	Review Comment
Hihara et al. [44] 2023	Compare HAp layer applied by powder jet vs. CaP paste that forms HAp in situ for dentin hypersensitivity	35 adults; split-mouth design; sensitivity assessed via VAS (air/scratch pain) over 12 weeks	Both groups showed similar improvement (air pain ~69%, scratch pain ~81%)	HAp reduced dentin hypersensitivity	Both HAp treatments reduced hypersensitivity; no significant difference between application types
Amaechi et al. [28] 2021	Assess whether HAp lotion enhances the effect of HAp toothpaste	30 adults; HAp toothpaste followed by either HAp or placebo lotion; 14-day treatment per phase using intraoral appliance with enamel blocks; mineral loss measured	Both groups showed significant remineralization; HAp lotion group showed greater effect (58.4% vs. 37.7%)	HAp toothpaste is effective; additional HAp lotion further enhances remineralization	HAp inhibits demineralization; additional HAp lotion boosts the effect
Hassani et al. [31] 2023	Compare the effect of plant-based vs. synthetic HAp in gum for remineralization	2 adults; Ca^2+^ and P content measured after chewing using enamel specimens in intraoral appliances	Synthetic HAp group showed significantly higher Ca^2+^ levels; plant-based HAp also increased Ca^2+^; no difference in P levels	HAp gum enhances enamel remineralization; synthetic HAp more effective than plant-based HAp	Synthetic HAp outperforms plant-based HAp in delivering Ca^2+^ for remineralization via chewing gum

(HAp: hydroxyapatite; CaP: calcium phosphate; VAS: Visual Analogue Scale, Ca^2+^: calcium; P: phosphorus).

## Data Availability

No new data were created or analyzed in this study. Data sharing is not applicable to this article.

## Abbreviations

The following abbreviations are used in this manuscript:

MIH	molar-incisor hypomineralization
Fl	fluoride
CPP-ACP	casein phosphopeptide–amorphous calcium phosphate
HAp	hydroxyapatite
